# *PIK3CA* Alterations in NSCLC: Clinical Characteristics of a “Neglected” Population of Oncogene-Addicted Patients

**DOI:** 10.3390/biomedicines14020362

**Published:** 2026-02-04

**Authors:** Sabrina Rossi, Arianna Pagliaro, Silvia Masini, Giovanna Finocchiaro, Luca Toschi, Emilio Bria, Vitale Antonio, Stefani Alessio, Alessandro Inno, Stefania Gori, Ettore D’Argento, Armando Santoro

**Affiliations:** 1 Medical Oncology and Hematology Unit, IRCCS Humanitas Research Hospital, 20089 Rozzano, Italy; sabrina.rossi@humanitas.it (S.R.); giovanna.finocchiaro@humanitas.it (G.F.); luca.toschi@humanitas.it (L.T.); armando.santoro@humanitas.it (A.S.); 2 Division of Oncology, Vizzolo Predabissi Hospital, ASST Melegnano e Martesana, 20077 Vizzolo Predabissi, Italy; arianna.pagliaro@asst-melegnano-martesana.it; 3 Department of Biomedical Sciences, Humanitas University, 20072 Pieve Emanuele, Italy; 4 U.O.C. Medical Oncology, Fondazione Policlinico Universitario Agostino Gemelli IRCCS, 00168 Rome, Italy; emilio.bria@policlinicogemelli.it (E.B.); antonio.vitale01@icatt.it (V.A.); alessio.stefani01@icatt.it (S.A.); ettore.dargento@policlinicogemelli.it (E.D.); 5 Department of Oncology, Sacro Cuore Don Calabria Hospital (IRCCS), 37024 Negrar, Italy; alessandro.inno@sacrocuore.it (A.I.); stefania.gori@sacrocuore.it (S.G.)

**Keywords:** precision oncology, PI3K/AKT/mTOR pathway, co-mutations

## Abstract

**Background/Objectives**: Alterations of the phosphatidylinositol 3-kinase catalytic subunit alpha gene (*PIK3CA*) are identified in approximately 2–4% of non-small cell lung cancer (NSCLC) cases; however, their biological and clinical relevance in NSCLC remains incompletely understood. This study aimed to comprehensively characterize the clinical and molecular features, as well as outcomes, of patients with *PIK3CA*-altered NSCLC across different disease stages. **Methods**: We conducted a retrospective multicenter analysis of 62 patients with histologically confirmed early-stage or advanced NSCLC-harboring *PIK3CA* alterations (mutations and/or gene amplifications) treated between 2015 and 2022 at three Italian institutions. Demographic, clinical, pathological, and molecular variables were systematically collected and analyzed. **Results**: *PIK3CA* mutations accounted for the majority of alterations (90.3%), while amplifications represented 9.7%. The most frequent mutations involved exon 9 (66.1%), predominantly E545K and E542K, followed by exon 20 (16.1%). Most patients were current or former smokers, and concomitant oncogenic alterations were detected in 59.7% of cases, most commonly *KRAS* mutations. A history of prior malignancy was reported in 24.6% of cases. In the metastatic setting, adenocarcinoma histology was associated with significantly longer overall survival (OS) compared with non-adenocarcinoma histologies (18.4 vs. 5.5 months; *p* = 0.02). Patients with PD-L1–negative tumors demonstrated a numerically longer OS than those with PD-L1–positive tumors; however, this difference did not reach statistical significance (19.1 vs. 5.4 months; *p* = 0.05). No statistically significant survival differences were observed according to specific *PIK3CA* mutation subtypes or treatment strategies. **Conclusions**: *PIK3CA*-altered NSCLC represents a molecularly heterogeneous and clinically understudied subgroup, frequently characterized by co-occurring oncogenic alterations. In this study, no definitive prognostic or predictive role for PIK3CA alterations could be established. Nevertheless, these findings provide a descriptive real-world characterization of this molecular subset and support the need for validation in larger, prospectively designed, molecularly stratified studies.

## 1. Introduction

The therapeutic success of precision oncology in non-small cell lung cancer (NSCLC) has been largely driven by the identification of actionable oncogenic drivers [1,2]. However, not all molecular alterations conform to the classical model of oncogene addiction. Among these, alterations in the phosphatidylinositol 3-kinase gene (*PIK3CA*) represent a distinct and relatively understudied molecular subgroup, frequently co-occurring with other driver alterations and raising questions about their pathogenic relevance and therapeutic implications [3,4,5]. Despite recent advances in targeted therapies and immunotherapy, NSCLC continues to be associated with high mortality worldwide [6]. In many patients with advanced NSCLC lacking actionable molecular alterations, chemotherapy remains a cornerstone of systemic treatment; however, its survival benefit is modest and it is frequently associated with clinically relevant toxicity [7]. Furthermore, no therapies are currently approved specifically for NSCLC-harboring *PIK3CA* alterations, highlighting the need to better understand the clinical and biological features of this molecular subgroup.

The PI3K family comprises multiple isoforms grouped into three classes, with Class I PI3Ks—including PI3Kα, β, γ, and δ—being the most relevant in solid tumors such as lung cancer [8,9]. Among them, PI3Kα, encoded by the *PIK3CA* gene, plays a central role in regulating cell growth, survival, metabolism, and angiogenesis [10]. Activating mutations or amplifications of *PIK3CA* lead to constitutive activation of the PI3K/AKT/mTOR pathway, promoting tumor progression through enhanced epithelial–mesenchymal transition (EMT), increased invasiveness, and resistance to tyrosine kinase inhibitors (TKIs) targeting upstream receptors such as EGFR [11,12]. In NSCLC, *PIK3CA* alterations, including point mutations and gene amplification, have been identified in a minority of patients, with mutations reported in approximately 2–4% of cases and amplifications in 12–18% [13,14,15,16]. These mutations cluster in two hotspot regions: the helical domain (exon 9, including E545K and E542K) and the kinase domain (exon 20, including H1047R and H1047L) [17]. Loss of *PTEN*, a negative regulator of this pathway, has also been described as a mechanism of PI3K pathway activation in lung cancer [18].

The clinical relevance of *PIK3CA* alterations has been clearly established in breast cancer, where they are detected in approximately 40% of hormone receptor–positive, HER2-negative cases [19]. In this setting, the PI3Kα-selective inhibitor alpelisib and the pan-AKT inhibitor capivasertib have demonstrated clinical benefit in phase III trials [20,21]. Accordingly, current international guidelines recommend routine testing for *PIK3CA* mutations in hormone receptor–positive, HER2-negative metastatic breast cancer, with assessment feasible on both tumor tissue and liquid biopsy samples [22,23].

Beyond its role in tumor growth, aberrant PI3K signaling supports metabolic reprogramming, enabling cancer cells to adapt to the hypoxic and nutrient-deprived tumor microenvironment, and promotes immune evasion through upregulation of PD-L1 and recruitment of immunosuppressive cells [18,24]. These mechanisms may contribute to resistance to immune checkpoint inhibitors and further highlight the role of the PI3K/AKT/mTOR axis in shaping the tumor microenvironment.

In NSCLC, *PIK3CA* alterations frequently coexist with other oncogenic drivers, such as *KRAS*, *EGFR*, and *BRAF*, raising questions about whether they function as independent oncogenic drivers or as co-mutations contributing to tumor heterogeneity and acquired resistance [5,17,25].

Despite increasing interest in this pathway, the clinicopathologic features and treatment outcomes of patients with *PIK3CA*-altered NSCLC remain poorly defined. In this study, we retrospectively analyzed a multicenter cohort of patients with early-stage and advanced NSCLC-harboring *PIK3CA* alterations to characterize their molecular landscape, co-mutation patterns, clinical features, and response to systemic therapies.

## 2. Materials and Methods

### 2.1. Patients Selection

This retrospective multicenter analysis included 62 patients with histologically confirmed NSCLC and documented *PIK3CA* alterations, including pathogenic mutations and/or gene amplifications. Patients were treated according to disease stage, molecular status (presence of targetable oncogenic alterations), programmed death-ligand 1 (PD-L1) expression, and clinician judgment in routine clinical practice at three Italian centers (IRCCS Humanitas Clinical and Research Center, IRCCS Fondazione Policlinico Universitario Agostino Gemelli, and IRCCS Sacro Cuore Don Calabria Hospital) between January 2015 and December 2022.

All patients were aged ≥ 18 years, and both early-stage and metastatic NSCLC cases were included. Baseline staging and follow-up imaging assessments primarily relied on thoracic and abdominal computed tomography (CT), performed according to routine clinical practice at each participating center, while positron emission tomography–computed tomography (PET-CT) and brain magnetic resonance imaging (MRI) were not routinely included in follow-up protocols but were performed selectively when clinically indicated.

Inclusion criteria were histologically confirmed NSCLC, documented *PIK3CA* mutation or amplification, availability of complete clinical and molecular data, and treatment administered at one of the participating centers. Exclusion criteria included lack of molecular profiling, incomplete clinical documentation, or insufficient follow-up for outcome assessment.

Patients with resectable locally advanced NSCLC underwent surgery with neoadjuvant or adjuvant chemotherapy, while those with unresectable locally advanced disease received definitive chemoradiotherapy or chemotherapy alone, based on multidisciplinary tumor board decisions. Patients with metastatic disease were treated with immunotherapy, chemoimmunotherapy, chemotherapy, or targeted therapy according to molecular profiling and PD-L1 expression levels. Treatment decisions were individualized based on patient age, disease stage, tumor histology, molecular profile, PD-L1 expression, comorbidities, and physician judgment, rather than being guided by a protocol-driven therapeutic algorithm. Owing to the retrospective design, detailed information regarding specific systemic drug names, radiotherapy dose and fractionation schedules, and treatment topography was not uniformly available across institutions.

### 2.2. Tissue Samples

Tumor tissue samples were obtained as part of routine diagnostic procedures at the participating institutions. Specimens included both biopsy and surgical resection samples, depending on disease stage and clinical context. One representative tumor sample per patient was used for molecular analyses. Tissue handling and processing followed local institutional standards of care. Consequently, some degree of inter-institutional variability in tissue handling and pre-analytical procedures cannot be excluded.

### 2.3. Molecular and Pathological Methods

Tumor samples were tested for molecular alterations (*EGFR*, *ALK*, *ROS1*, *KRAS*, *BRAF*, *HER2*, *MET*, *RET*, and *PIK3CA*) using locally validated, clinically accredited molecular diagnostic assays routinely employed at each institution, including polymerase chain reaction (PCR)-based methods and/or next-generation sequencing (NGS) panels, in accordance with national diagnostic standards. In squamous cell carcinoma cases, molecular testing was performed only in never-smokers, in accordance with clinical guidelines. Testing methodologies varied across centers and over time according to institutional practice and technological availability. Due to the retrospective design, detailed information on the specific molecular techniques used (e.g., PCR-based assays vs. NGS panels) was not uniformly available, and this variability may have influenced analytical sensitivity and gene coverage across institutions.

### 2.4. Statistical Analysis

The study aimed to describe the clinical and tumor characteristics of patients with NSCLC-harboring *PIK3CA* alterations. Categorical variables were summarized as counts and percentages, while continuous variables were described using mean, median, and range.

Time to progression (TTP), overall survival (OS), and progression-free survival (PFS) were estimated using the Kaplan–Meier method. Differences between patient subgroups were evaluated using the log-rank test. TTP was calculated from the date of initial diagnosis to first documented disease recurrence, OS from the date of diagnosis to death from any cause, and PFS from treatment initiation to disease progression or death, whichever occurred first.

All statistical analyses were performed using SAS version 9.4 (SAS Institute, Cary, NC, USA). Statistical significance was defined as a two-sided *p* value <0.05.

## 3. Results

### 3.1. Patients Characteristics

In this retrospective series, 62 patients with *PIK3CA* mutations (*n* = 56; 90.3%) or amplifications (*n* = 6; 9.7%) were included. The median age was 71 years (range, 47−85); approximately half of the patients were male (54.8%), and 19.3% were never-smokers. Overall, 16.1% of patients had squamous histology; however, molecular testing in this subgroup was performed only in never-smokers. Approximately one-quarter of the cohort had a history of prior malignancies, including two patients with hematologic cancers. Patient characteristics are summarized in Table 1.

The most frequent *PIK3CA* mutation subtypes involved exon 9 (66.1%), particularly E545K (41.9%) and E542K (22.6%), followed by exon 20 (16.1%), mainly H1047R (8.0%). In two cases (3.2%) the specific *PIK3CA* mutation subtype was not reported in the medical records. The distribution of *PIK3CA* mutation types is reported in Table 2.

Co-mutations were identified in 59.7% of patients (*n* = 37). The most frequent was a *KRAS* co-mutation, observed in 41.9% of cases, with the *KRAS* G12C subtype present in 27.4% of patients. Sensitizing *EGFR* mutations were detected in 9.7% of cases, and *BRAF* V600E co-mutations in 4.8%. In addition, one *ROS1* fusion and one *MET* exon 14 skipping mutation were identified. PD-L1 status was assessed in 90.3% of patients and was ≥50% in 10 cases, 1−49% in 20 cases, and <1% in 26 patients.

At diagnosis, 4 patients presented with stage I NSCLC (6.5%), 10 with stage II (16.1%), 9 with stage IIIA (14.5%), 4 with stage IIIB (6.5%), and 35 with metastatic disease (56.5%). One-third of the patients underwent surgery, and 16.1% received neoadjuvant or adjuvant treatment. Among patients with de novo or relapsed metastatic NSCLC, metastatic sites at diagnosis included the brain (25.9%), pleura or lung (61.1%), and bone (29.6%). Approximately one-quarter of patients had a high tumor burden at diagnosis, defined as ≥ 3 metastatic sites. First-line treatment consisted of single-agent immunotherapy in 9.2% of patients, chemoimmunotherapy in 18.5%, chemotherapy in 27.8% (13 patients received platinum-based doublets and 2 received single-agent chemotherapy) and targeted therapies in 13.0% of cases.

Overall, *PIK3CA*-altered NSCLC in this cohort was characterized by a predominance of smokers, frequent co-occurring oncogenic alterations—particularly *KRAS* mutations—and a high proportion of patients presenting with advanced disease at diagnosis.

### 3.2. Clinical Outcomes and Survival

The median follow-up at the time of data analysis was 36.7 months. Survival analyses were conducted separately for patients with early-stage/locally advanced disease and for those with metastatic disease at diagnosis or at relapse. Among patients with early-stage and locally advanced NSCLC, 19 of 27 (70.4%) experienced disease relapse or progression: 3 patients with stage I disease (all treated with surgery), 9 with stage II (all treated with surgery, including 5 who received adjuvant chemotherapy), 5 with stage IIIA (all treated with surgery, including 3 who received neoadjuvant chemotherapy), and 2 with stage IIIB (both treated with sequential chemoradiotherapy).

The median TTP was 12.3 months (95% CI: 4.0−36.0) for stage I patients, 13.5 months (95% CI: 10.6−18.5) for stage II, 13.9 months (95% CI: 10.8−44.8) for stage IIIA, and 6.1 months (95% CI: 6.1−7.6) for stage IIIB. The median OS was 41.1 months (95% CI: 5.4−67.4) for stage I patients, 38.3 months (95% CI: 15.9−52.4) for stage II, 31.1 months for stage IIIA (95% CI: 24.5−31.1), 10.5 months for stage IIIB (95% CI: 10.5−11.5).

**Figure 1:**
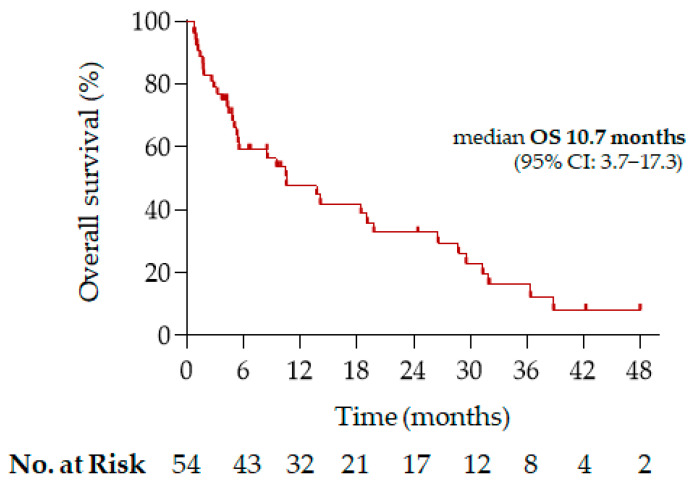
Kaplan–Meier curve for overall survival (OS) in patients with metastatic *PIK3CA*-altered NSCLC.

Among patients with metastatic disease at diagnosis (*n* = 35) and those who later developed distant metastases (*n* = 19), the median OS for the overall metastatic population was 10.5 months (95% CI: 3.7−17.3) (Figure 1).

**Figure 2:**
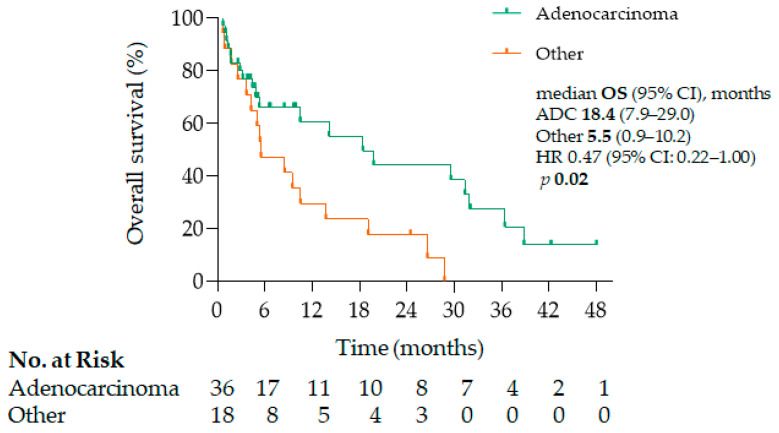
Kaplan–Meier curves for overall survival (OS) by histology in patients with metastatic *PIK3CA*-altered NSCLC. Abbreviations: ADC, adenocarcinoma.

Female patients had a longer median OS than male patients; however, this difference was not statistically significant (14.1 months vs. 5.4 months; *p* = 0.51). Patients with adenocarcinoma histology showed a significantly longer OS compared with those with other histologic subtypes (*p* = 0.02) (Figure 2).

A longer OS was also observed in patients with a lower tumor burden (defined as< 3 metastatic sites), although this difference did not reach statistical significance (*p* = 0.23). No differences in OS were observed according to age (≥75 vs. <75 years, *p* = 0.83), smoking status (current or former smokers vs. never smokers, *p* = 0.36), or the presence of brain metastases at diagnosis (*p* = 0.92).

Regarding tumor molecular characteristics, a longer, although not statistically significant, OS was observed in patients harboring co-mutations (18.4 vs. 8.5 months; *p* = 0.59). No differences were found in patients with concurrent *KRAS* mutations (*p* = 0.57), including the *KRAS* G12C subtype (*p* = 0.37). Among *PIK3CA* mutation subtypes, exon 9 E545K was associated with a clinically meaningful, though not statistically significant, longer OS (19.8 vs. 8.5 months; *p* = 0.62). PD-L1-negative patients (*n* = 16) had a longer OS compared with those with PD-L1 ≥ 1% tumors (*n* = 32), although this difference did not reach statistical significance (19.1 vs. 5.4 months; HR 0.45, 95% CI: 0.22−0.94; *p* = 0.05) (Table 3).

Treatment-specific survival outcomes are reported descriptively due to the small number of patients within each treatment subgroup. Among patients who received first-line treatment for metastatic disease, the median PFS was 4.0 months (95% CI: 2.6−12.0) with chemotherapy, 27.5 months (95% CI: 14.3−NR) with single-agent immunotherapy, 6.0 months (95% CI: 2.3−6.1) with chemoimmunotherapy, and 6.7 months (95% CI: 5.8−6.7) with targeted therapies (*p* = 0.07). The corresponding median OS values were 5.5 months (95% CI: 4.7−36.4), 29.6 months (95% CI: 19.8−32.0), 10.5 months (95% CI: 4.4−13.7), and 31.3 months (95% CI: 14.1−NR), respectively (*p* = 0.38) (Table 4).

Outcomes are reported according to the type of systemic therapy received. However, due to the small number of patients within each subgroup and the heterogeneity of clinical settings, no formal comparative analyses across these treatment categories were performed.

In summary, survival outcomes were heterogeneous and largely driven by disease stage and histology, while no statistically significant differences emerged according to specific *PIK3CA* mutation subtypes or treatment categories.

## 4. Discussion

This study provides insight into the clinical and molecular characteristics of NSCLC patients harboring *PIK3CA* alterations, a relatively rare and understudied molecular subgroup. Although based on a limited cohort, our analysis contributes to the growing body of literature investigating the clinical relevance of *PIK3CA* alterations in NSCLC. Given its retrospective multicenter design, the present study is inherently subject to selection bias, incomplete data capture, and heterogeneity in diagnostic procedures and therapeutic approaches across participating institutions. These methodological constraints should be considered when interpreting the findings.

In our cohort, *PIK3CA* alterations, either mutations or amplifications, were more frequently observed in older patients (median age, 71 years), current or former smokers, and tumors with low PD-L1 expression (<1%). No significant sex-related differences were observed. The most common mutations involved exon 9 (E545K, E542K) and exon 20 (H1047R), and frequently co-occurred with other actionable driver alterations, particularly *KRAS*, *EGFR*, and *BRAF V600E*. More than half of patients had metastatic disease at diagnosis, with frequent involvement of the brain, pleura or lung, and bone. A notable proportion (~25%) had a history of prior malignancies, which may suggest a broader role of *PIK3CA* alterations in tumorigenesis.

These findings are broadly consistent with those reported by Scheffler et al., who described 42 patients with *PIK3CA*-mutated NSCLC. Similar to our cohort, they observed a high prevalence of smokers and co-occurring driver alterations (57.1%), as well as a predominance of exon 9 mutations, particularly E545K. However, they reported a higher frequency of *PIK3CA* mutations in squamous cell carcinoma compared with adenocarcinoma (8.9% vs. 2.9%, *p* < 0.001) [17]. This discrepancy may be explained by differences in molecular testing strategies, as in our setting, molecular profiling is routinely performed in adenocarcinomas and in squamous cell carcinomas only in never-smokers, potentially introducing selection bias.

Historically, the prognosis of surgically resected NSCLC has been strongly associated with TNM stage, with 5-year survival rates ranging from 90% in stage IA to 12% in stage IIIC [26]. Locoregional recurrence represents a common pattern of failure after surgery, with reported rates ranging from 5–19% in stage I to 24–40% in stage IIIA [27]. In our cohort, disease relapse or progression occurred in 70.4% (19/27) of early-stage and locally advanced cases, with median TTP ranging from 12.3 to 13.9 months in stages I–IIIA and decreasing to 6.1 months in stage IIIB. Although limited by sample size and the absence of a matched *PIK3CA* wild-type control cohort, these findings may suggest a more aggressive clinical course in patients harboring *PIK3CA* alterations.

Data from a previously published series provide a similarly heterogeneous pattern, and in most cohorts, *PIK3CA* alterations were not associated with a statistically significant difference in overall survival. In the study by Scheffler et al., which included both early- and advanced-stage disease, overall survival did not differ between operable *PIK3CA*-mutant patients and a matched control group [17]. Among inoperable cases, a non-significant trend toward shorter survival was observed in the *PIK3CA*-mutant group compared with the overall non-operated control population. Notably, significantly longer overall survival was reported only in the *EGFR*-mutant subgroup when compared with *PIK3CA*-mutant patients, while no other molecular subgroup demonstrated a significant survival difference. Consistent with these findings, Chaft et al. reported shorter overall survival in patients with *PIK3CA* mutations and concurrent oncogenic driver alterations compared with those with *PIK3CA* mutations alone [25]. Analyses of large genomic cohorts have suggested a possible association between *PIK3CA* mutations and poorer survival in lung adenocarcinoma; however, this finding was not consistently observed across different datasets [28]. Additional matched retrospective analyses likewise failed to demonstrate an independent prognostic impact of *PIK3CA* alterations [29].

Collectively, the available evidence indicates that survival outcomes in *PIK3CA*-altered NSCLC are heterogeneous and do not support a consistent independent prognostic role for *PIK3CA* alterations.

Data on treatment response in *PIK3CA*-altered NSCLC remain limited and largely descriptive. In the cohort reported by Scheffler et al., therapeutic strategies were heterogeneous, and a clinical benefit with targeted or anti-angiogenic combinations was reported only in a small number of cases, while responses to platinum-based chemotherapy were not consistently documented [17]. Among patients receiving systemic therapy, reported median overall survival in advanced-stage disease was approximately one year. Overall, these findings do not support a clear predictive role for *PIK3CA* alterations and suggest that treatment outcomes are more likely influenced by co-occurring molecular drivers and clinical factors than by *PIK3CA* status alone. Although we observed numerical differences in survival according to the type of first-line systemic therapy, these were not statistically significant and should be interpreted with caution given the limited sample size. In particular, no consistent evidence of differential sensitivity to immunotherapy or TKIs emerged in relation to *PIK3CA* status. Therefore, this study was not designed to assess treatment efficacy according to molecular subtype, and no definitive conclusions can be drawn regarding the predictive role of *PIK3CA* alterations. Future prospective studies with molecularly stratified cohorts are needed to clarify potential interactions between *PIK3CA* alterations and systemic treatment outcomes.

In our study, *PIK3CA* alterations frequently co-occurred with other oncogenic drivers—most commonly *KRAS* and *EGFR* mutations—consistent with previously published genomic analyses [5,17,25,28]. This pattern suggests that *PIK3CA* may often act as a cooperating rather than initiating event in tumorigenesis, and its prognostic or predictive relevance is likely influenced by the broader mutational context.

Notably, *PIK3CA* mutations have also been implicated as a potential mechanism of resistance to targeted therapies in NSCLC, particularly in tumors harboring other driver alterations such as *EGFR* mutations, *KRAS* G12C, and *MET* exon 14 skipping [30,31,32,33,34,35]. In these settings, the PI3K pathway may sustain proliferative signaling and bypass the inhibitory effects of targeted agents, contributing to both primary and acquired resistance.

Despite recent advances in molecular oncology, therapeutic strategies targeting the PI3K/AKT/mTOR pathway have not yet translated into clear clinical benefit for patients with NSCLC. While PI3K inhibitors have demonstrated promising activity in other tumor types, such as breast cancer, their efficacy in NSCLC remains limited [36]. Although preclinical models suggest that *PIK3CA*-mutant tumors may be sensitive to PI3K inhibition, subsequent clinical trials in NSCLC have not confirmed a consistent therapeutic benefit [37,38,39,40,41]. No patients in our cohort received PI3K pathway inhibitors, and the above considerations refer to findings reported in the literature. Taken together, these observations underscore the complexity of therapeutic targeting in lung cancer, where treatment selection is increasingly informed by molecular biomarkers and combination strategies aimed at overcoming resistance mechanisms [42].

Overall, this study expands current knowledge of the clinical and molecular landscape of *PIK3CA*-altered NSCLC, highlighting key associations with co-mutation patterns, smoking history, and adverse outcomes in early-stage disease. However, the clinical implications of our findings remain hypothesis-generating and should be interpreted within the context of the study limitations. Accordingly, the results should be viewed primarily as a descriptive characterization of a rare molecular subgroup in real-world clinical practice rather than as a definitive prognostic or predictive analysis. While the role of *PIK3CA* as an independent oncogenic driver or resistance mediator remains debated, our results support the need for larger, controlled, and molecularly stratified studies to better define its prognostic and therapeutic relevance. In addition, effective targeted therapies remain lacking, as PI3K inhibitors have shown limited clinical benefit to date. Thus, the optimal management of this subgroup remains an unmet clinical need warranting further investigation.

### Limitations

This study has several limitations that should be considered when interpreting the findings. First, the retrospective design inherently carries the risk of selection bias and may have contributed to variability in diagnostic procedures and therapeutic strategies across participating centers. Owing to the retrospective multicenter design, imaging schedules and follow-up assessments were not fully standardized across centers, resulting in potential inter-center heterogeneity in imaging strategies. In addition, reliance on medical records may have resulted in incomplete capture of certain clinical variables.

The relatively small sample size represents a major limitation, reducing statistical power and limiting the ability to detect prognostic or predictive effects, particularly in subgroup analyses. The low number of survival events also limited the feasibility of multivariable survival analysis.

An additional major limitation is the absence of a matched *PIK3CA* wild-type control cohort, which precludes definitive conclusions regarding the independent prognostic significance of *PIK3CA* alterations. The frequent coexistence of other oncogenic drivers in this population further complicates interpretation, as observed outcomes may reflect the broader molecular context rather than the isolated contribution of *PIK3CA* status.

Moreover, treatment heterogeneity across disease stages and lines of therapy limits the interpretability of treatment-specific outcomes and precludes a formal assessment of predictive value. Systemic therapy choices and radiotherapy approaches were individualized rather than protocol-driven, and detailed information on specific drug regimens, radiation dose, and treatment fields was not consistently available across centers.

Finally, molecular characterization was restricted to routinely available clinical assays. Data on mutation clonality, variant allele frequency, and comprehensive downstream PI3K pathway alterations were not consistently available, precluding a more refined biological stratification of *PIK3CA*-altered tumors. In addition, detailed information on the specific molecular testing methodologies used across centers was not systematically collected.

## 5. Conclusions

NSCLC-harboring *PIK3CA* alterations represents a heterogeneous and understudied molecular subgroup. This study provides a descriptive overview of the clinical and molecular characteristics of NSCLC-harboring PIK3CA alterations in a real-world multicenter setting. In our cohort, these alterations were associated with frequent co-mutations, smoking history, and low PD-L1 expression. In early-stage disease, *PIK3CA* mutations appeared to correlate with shorter time to progression, although their definitive prognostic significance remains unclear. In the metastatic setting, survival outcomes and treatment responses were heterogeneous, and no consistent differences emerged across systemic therapy types.

These findings should be interpreted within the context of the study limitations, particularly the small sample size and the absence of a matched PIK3CA wild-type control cohort. Nevertheless, they provide preliminary insights that warrant further investigation in larger, prospective, and molecularly stratified cohorts.

## Figures and Tables

**Table 1:** Patient characteristics.

	*N =* 62
Sex (Male/Female), *n* (%)	34/28 (54.8/45.2)
Median age, years (range)	71 (47−85)
Smoking history, *n* (%)	
Never	12 (19.3)
Current	30 (48.4)
Former	20 (32.3)
Prior malignancies, *n* (%)	15 (24.2)
Histology, *n* (%)	
Adenocarcinoma	42 (67.8)
Squamous cell	10 (16.1)
Other	10 (16.1)
*PIK3CA* mutation, *n* (%)	
E542K	14 (22.6)
E545K	26 (41.9)
H1047R	5 (8.0)
Other	9 (14.5)
Not assessed	2 (3.2)
*PIK3CA* amplification, *n* (%)	6 (9.7)
Co-mutations, *n* (%)	
*KRAS/KRAS G12C*	26/17 (41.9/27.4)
*EGFR*	6 (9.7)
*BRAF V600E*	3 (4.8)
*ROS1*	1 (1.6)
*MET*	1 (1.6)
PD-L1, *n* (%)	
<1%	26 (41.9)
1−49%	20 (32.2)
≥50%	10 (16.1)
Not assessed	6 (9.7)
Metastatic sites, *n* (%)	
Central nervous system	14/54 (25.9)
Pleura/lung	33/54 (61.1)
Bone	16/54 (29.6)
Other	18/54 (33.3)
Number of metastatic sites ≥ 3, *n* (%)	16/54 (29.6)
Staging at diagnosis, *n* (%)	
I/II	4/10 (6.5/16.1)
IIIA	9 (14.5)
IIIB/IIIC	4/0 (6.5/0.0)
IV	35 (56.5)
Surgery, *n* (%)	21 (33.9)
Adjuvant/neoadjuvant therapy, *n* (%)	10 (16.1)
First-line treatment, *n* (%)	
Chemotherapy	15/54 (27.8)
Immunotherapy	5/54 (9.2)
Chemoimmunotherapy	10/54 (18.5)
Target therapy	7/54 (13.0)

**Table 2:** Types of *PIK3CA* mutations.

Type of Mutation	*n* (%)
Exon 9	41 (66.1)
E545K	26 (41.9)
E542K	14 (22.6)
I459V	1 (1.6)
Exon 20	10 (16.1)
H1047R	5 (8.0)
H1047L	1 (1.6)
M1043I	2 (3.2)
M1004I	1 (1.6)
N1044K	1 (1.6)
Exon 2	3 (4.8)
P104L	1 (1.6)
R115L	1 (1.6)
R93P	1 (1.6)
Exon 5	2 (3.2)
V344E	1 (1.6)
D352H	1 (1.6)
Exon 13	1 (1.6)
E726K	1 (1.6)

**Table 3:** Univariate analysis for overall survival (OS).

	OS in Metastatic PIK3CA-Altered Patients (*n* = 54), Months	*p*
Sex (Male/Female)	5.4 vs. 14.1 (HR 0.80; 95% CI: 0.41−1.55)	0.51
Age (≥75 vs. <75 years)	19.8 vs. 10.5 (HR 0.93; 95% CI: 0.48−1.81)	0.83
Smoking history (current/former vs. never)	10.5 vs. 14.1 (HR 0.70; 95% CI: 0.34−1.15)	0.36
Histology (adenocarcinoma vs. other)	18.4 vs. 5.5 (HR 0.47; 95% CI: 0.22−1.00)	0.02
Brain metastases at baseline (Yes/No)	10.5 vs. 13.7 (HR 0.96; 95% CI: 0.45−2.07)	0.92
Number of metastatic sites (≥3 vs. <3)	8.5 vs. 18.4 (HR 0.66; 95% CI: 0.32−1.38)	0.23
Concomitant mutations (Yes/No)	18.4 vs. 8.5 (HR 0.84; 95% CI: 0.43−1.64)	0.59
*KRAS* mutations (Yes/No)	10.5 vs. 13.7 (HR 0.83; 95% CI: 0.42−1.62)	0.57
*KRAS* G12C mutations (Yes/No)	10.5 vs. 13.7 (HR 0.71; 95% CI: 0.32−1.59)	0.37
*EGFR* mutations (Yes/No)	31.4 vs. 9.5 (HR 0.66; 95% CI: 0.20−2.26)	0.51
*PIK3CA* E545K (Yes/No)	19.8 vs. 8.5 (HR 0.85; 95% CI: 0.44−1.64)	0.62
*PIK3CA* E542K (Yes/No)	8.5 vs. 13.7 (HR 0.85; 95% CI: 0.38−1.72)	0.48
PD-L1 ≥ 1% vs. <1%	5.4 vs. 19.1 (HR 0.45; 95% CI: 0.22−0.94)	0.05

**Table 4:** Univariate analysis for progression-free survival (PFS) and overall survival (OS) according to treatment received.

First-Line Treatment	*n*	Median PFS, Months	Median OS, Months
Chemotherapy	15	4.0 (95% CI: 2.6−12.0)	5.5 (95% CI: 4.7−36.4)
Chemoimmunotherapy	10	6.0 (95% CI: 2.3−6.1)	10.5 (95% CI: 4.4−13.7)
Immunotherapy	5	27.5 (95% CI: 14.3−NR)	29.6 (95% CI: 19.8−32.0)
Targeted therapies	7	6.7 (95% CI: 5.8−6.7)	31.3 (95% CI: 14.1−NR)

## Data Availability

The original contributions presented in this study are included in the article. Further inquiries can be directed to the corresponding author.
